# Ten-Year Results of the Triathlon Knee Replacement: A Cohort Study

**DOI:** 10.7759/cureus.15211

**Published:** 2021-05-24

**Authors:** Vikki Wylde, Chris Penfold, Alice Rose, Elizabeth Bradshaw, Michael R Whitehouse, Ashley W Blom

**Affiliations:** 1 Musculoskeletal Research Unit, University of Bristol, Bristol, GBR

**Keywords:** knee replacement, cohort study

## Abstract

Introduction

Studies evaluating the outcomes of different brands of knee prostheses are important to monitor patient outcomes and generate evidence to aid decisions around the choice of implant. The Triathlon® prosthesis (Stryker, Limerick, Ireland), one of the most commonly used total condylar knee prosthesis, is designed to provide greater knee motion and the potential for longer implant survivorship. The aim of this cohort study was to evaluate outcomes and survivorship of the Triathlon total knee replacement (TKR) up to 10 years post-operative.

Methods

Two-hundred sixty-six (266) patients listed for a Triathlon TKR in one orthopaedic hospital were recruited. Assessments were conducted preoperatively and then at three months and one, two, three, five, seven, and 10 years after surgery. Outcomes assessed included pain, function, knee-related quality of life (QoL), satisfaction, kneeling ability, activity levels, American Knee Society Score, complications, and survivorship.

Results

Large improvements in patient-reported outcomes were observed in the first three months after surgery, followed by small improvement up to one year post-operative, and then outcomes plateaued up to 10 years post-operative. Satisfaction with overall outcome ranged from 79%-94% over the duration of follow-up. Activity levels and kneeling ability were similar before and after surgery. There was a large improvement in the median American Knee Society score in the first three months post-operative, followed by a small but gradual improvement to 10 years post-operative. Survivorship was 95.4% (95% confidence interval 91.8-97.5%) at 10 years post-operative.

Conclusions

This study found that the Triathlon TKR results in excellent outcomes and survivorship to 10 years post-operative.

## Introduction

Primary total knee replacement (TKR) is one of the most common elective surgical procedures, with over 100,000 operations performed in the National Health Service (NHS) each year [1-2]. Traditionally, TKR was performed to relieve chronic pain and restore basic function to allow patients to return to their activities of daily living. While these are still the most common reasons patients elect to have TKR, patient expectations of TKR are expanding to include more demanding activities such as sports, leisure activities, physically demanding jobs and kneeling [3-4]. Although many patients have good outcomes after TKR, approximately 20%-30% of patients report long-term pain, functional limitations and dissatisfaction with the outcome of their surgery [5-7]. Also, difficulty with more challenging activities, such as kneeling, are common [8]. To meet the growing expectations of patients and to optimise outcomes, manufacturers are continually developing new prosthetic designs.

In 2019, the Triathlon® prosthesis (Stryker, Limerick, Ireland) was the second most commonly used total condylar knee prosthesis recorded in the National Joint Registry, accounting for 20% of TKRs [1]. The Triathlon prosthesis was designed to improve on prior designs offered by the manufacturer, including a single radius design allowing for mid-flexion stability, to give great knee motion and longer implant survivorship [9]. The Triathlon TKR has been found to result in better outcomes than predecessor implants including the Duracon (Stryker) [10] and Kinemax (Stryker) [11]. Despite the widespread use of the Triathlon TKR, few studies have conducted a comprehensive evaluation of longer-term outcomes after TKR using a longitudinal study design [12-16]. Such studies are required to develop a robust evidence base on the outcomes of the Triathlon TKR. The aim of this prospective cohort study was to evaluate patient-reported outcomes, clinical outcomes and survivorship of the Triathlon TKR up to 10 years post-operative. The hypothesis was that the Triathlon TKR would result in good patient outcomes and survivorship up to 10 years post-operative.

## Materials and methods

Patients

This was a single-centre, prospective cohort study conducted at one regional elective orthopaedic centre in the South West of England [12,17]. Reporting follows recommendations from the STROBE (STrengthening the Reporting of OBservational studies in Epidemiology) initiative. Between October 2006 and October 2009, consecutive patients listed for a primary Triathlon TKR because of osteoarthritis with one of 11 participating consultant orthopaedic surgeons were invited to participate in the study. Patients who were undergoing revision surgery or who could not complete the questionnaires in the English language were excluded. Patients who were interested in participating were asked to provide informed, written consent. Ethics approval for the study was received from Southmead NHS Research Ethics Committee (Reference: 06/Q2002/80).

Methods of assessment

Patients were assessed pre-operatively and then at regular intervals post-operatively: three months, one year, two years, three years, five years, seven years, and 10 years. The assessment included patient-reported outcome measures, clinical examination by a trained physiotherapist and review of medical records. All these assessments were performed at each time point, with the exception of the assessment at two years and seven years, which was by postal questionnaire and did not involve a clinical assessment. Non-responders at each timepoint were sent a single reminder questionnaire.

Patient-reported outcome measures

Knee symptoms were assessed using the Western Ontario McMasters University Osteoarthritis Index (WOMAC) [18] and Knee Injury and Osteoarthritis Outcome Score (KOOS) knee-related quality of life subscale [19]. The WOMAC pain, function and stiffness subscales assess the knee pain severity during five activities, functional limitations experienced when performing 17 tasks, and the degree of knee stiffness in the morning and later in the day, with each subscale scored 0-100 (worst to best). The KOOS knee-related quality of life subscale is a four-item questionnaire assessing patients’ awareness of their knee problem and impact on daily life, with a score of 0-100 (worst to best). Satisfaction was assessed with the Patient Satisfaction Scale [20], a four-item questionnaire assessing satisfaction with pain relief, ability to do daily activities, ability to do leisure activities and overall outcome, with response options of ‘very satisfied’, ‘somewhat satisfied’, ‘somewhat dissatisfied’ and ‘very dissatisfied’. Activity level was measured using the University of California at Los Angeles (UCLA) Activity Score [21], which is scored from 0-10 (low to high activity level). Single-item questions were used to ask participants about difficulty kneeling, improvement in quality of life and whether they regretted having their knee replaced. In the pre-operative questionnaire, participants were also asked questions about sociodemographic characteristics, number of medical co-morbidities using the Self-Administered Comorbidities questionnaire [22] and number of painful joints.

American Knee Society Score

A trained research physiotherapist conducted a knee assessment during a research appointment at the hospital or a home visit to participants. The assessment included knee stability, range of motion, alignment and pain. The American Knee Society Score (AKSS) knee score [23] was calculated, ranging from 0-100 (worst to best).

Complications and survivorship

The research physiotherapist asked participants about any surgical and medical complications during each clinical assessment. Self-reported complications were then confirmed through a review of participants’ medical records. Revision surgery, including dates and indications, were extracted and used to calculate survivorship. Deceased patients were identified from hospital records and the date and cause of death were recorded.

Statistical analysis

Participants’ sociodemographic characteristics were summarised using the median and interquartile range (IQR) or percentage. Median WOMAC, KOOS, UCLA, and AKSS knee scores were plotted on box and whisker plots across the 10-year follow-up based on participants with all available data available at each time point. The bold line in the box and whisker plot is the median, the outer edges of the boxes are the lower (25%) and upper (75%) quartiles, and the whisker covers the range of the data excluding outliers (>1.5x interquartile range). Satisfaction and kneeling ability are reported using percentages. Best-case survivorship curves were calculated (1-Kaplan-Meier estimates) with failure defined as all-cause revision, and patients who withdrew or died were considered as having implants that remained in situ but censored at those time points.

## Results

Participants

**Figure 1:**
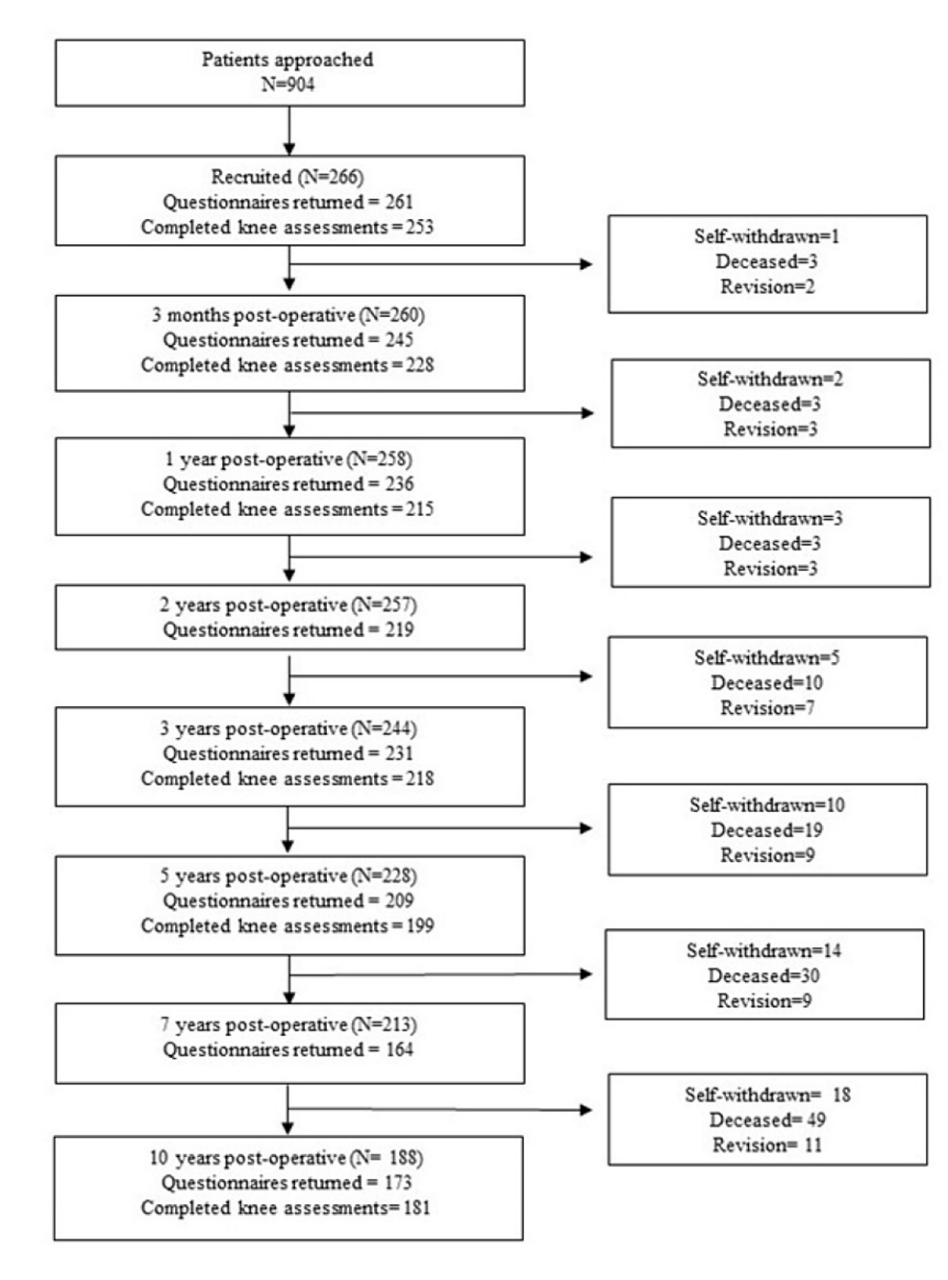
Participant flow

Over the three year recruitment period, 904 patients listed for a primary Triathlon TKR were invited to take part in the study, and 266 patients (29%) agreed to participate. The participants’ baseline sociodemographic characteristics are displayed in Table 1. Participants and non-participants had a similar median age (70 years (IQR 62-77) vs 72 years (IQR 64-79), respectively) and the same percentage of females (64%). The surgical approaches used were medial parapatellar (66%), medial subvastus (33%) and lateral parapatellar (1%). Most participants (92%) received a cruciate retaining prosthesis. An overview of participant figures at each assessment time point, along with reasons for loss to follow-up, are provided in Figure 1.

**Table 1:** Participants sociodemographic characteristics BMI: body mass index

Variable	Median (IQR) or number (%)
Age in years	70 (62-77)
Female	169 (64%)
BMI	30 (27-35)
Number of co-morbidities	2 (1-3)
Number of painful joints	4 (2-5)
White ethnicity	252 (98%)
Post-secondary education	76 (29%)
Married/cohabiting	171 (66%)
Retired	180 (70%)

Pain, function, stiffness and quality of life

**Figure 2:**
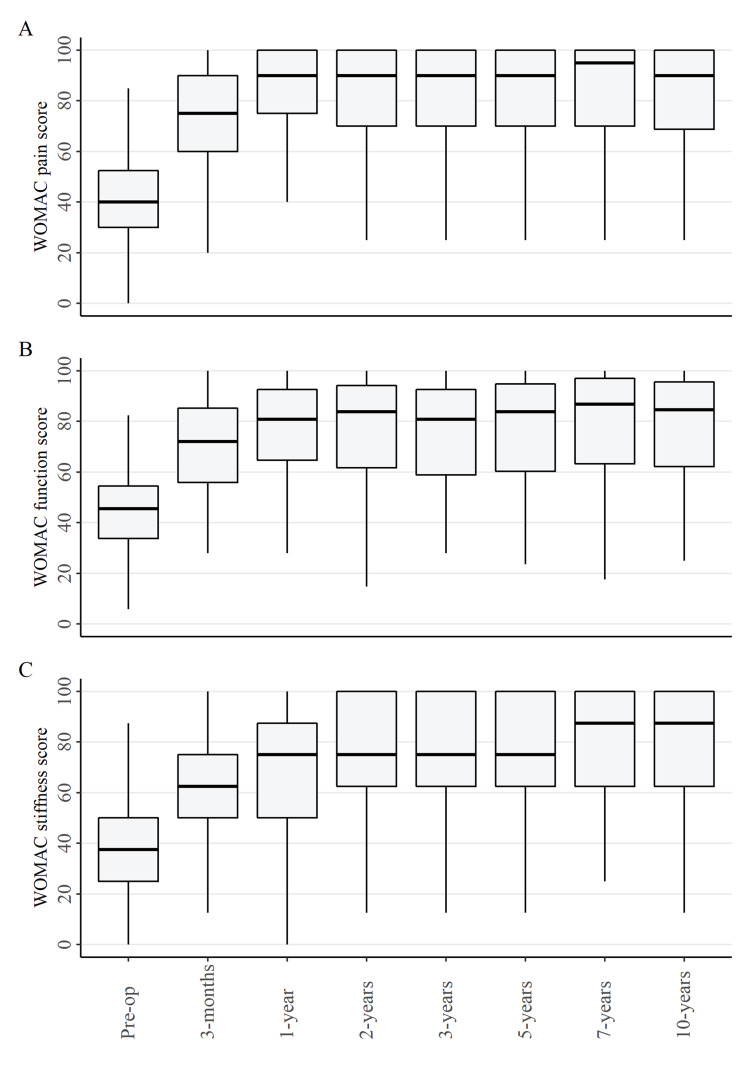
WOMAC scores for A) pain scale, B) function scale, and C) stiffness scale WOMAC: Western Ontario McMasters University Osteoarthritis Index

**Figure 3:**
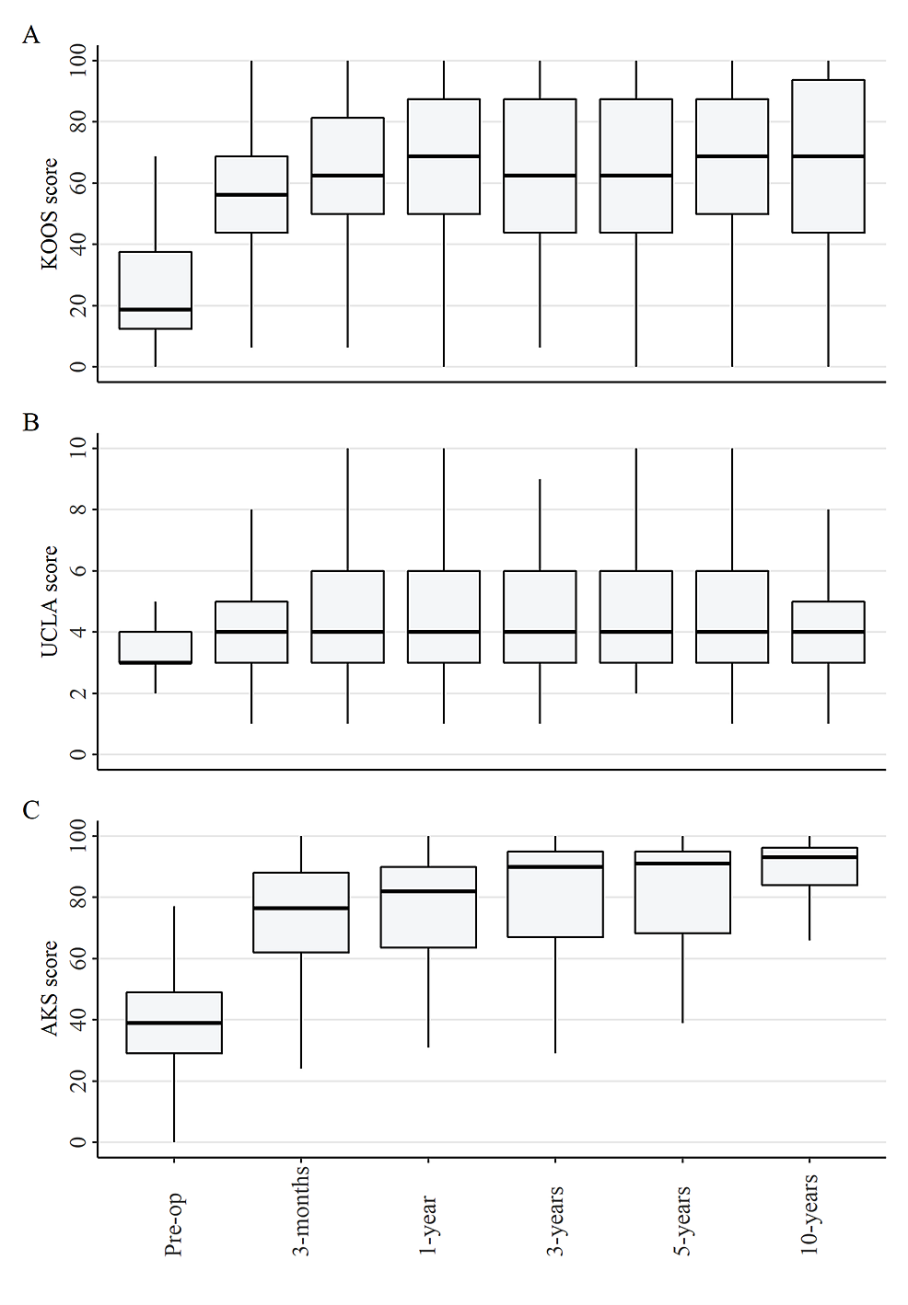
A) KOOS B) UCLA Activity score C) AKSS score KOOS: Knee Injury and Osteoarthritis Outcome Score; UCLA: University of California Los Angeles; AKSS: American Knee Society Score

WOMAC scores for pain, function and stiffness over the 10-year follow-up period are displayed in Figures 2A-C and KOOS knee-related quality of life scores in Figure 3, panel A. For all outcomes, the greatest improvement occurred in the first three months after surgery. Median outcome scores continued to demonstrate smaller improvements up to one year post-operative and then outcomes stabilised up to 10 years post-operative. The percentage of patients who reported a large improvement in their quality of life at each assessment time (defined as a response of ‘a great improvement’ or ‘more than I dreamed possible’) ranged from 60% to 77% over the duration of follow-up.

Satisfaction

The percentage of patients who were satisfied (somewhat or very) with the four aspects of outcome assessed in the Patient Satisfaction Scale at each follow-up point are presented in Table 2. Between 79%-94% of patients were satisfied with their outcomes over the follow-up duration. Pain relief had the highest proportion of satisfied patients at all time points (89-94%) and ability to do leisure activities the lowest proportion (79-89%). The percentage of patients who regretted having their knee replaced ranged from 3% at three months post-operative to 9% at 10 years post-operative.

**Table 2:** Percentage of patients who were somewhat or very satisfied with their outcome at each follow-up assessment time

	Pain relief	Ability to do daily activities	Ability to do leisure activities	Overall outcome
3 months	94%	87%	86%	92%
1 year	94%	92%	89%	91%
2 years	90%	84%	82%	89%
3 years	89%	84%	82%	85%
5 years	90%	83%	80%	86%
7 years	94%	87%	82%	90%
10 years	89%	85%	79%	88%

Activity levels

Median UCLA activity scores over the follow-up period are displayed in Figure 3, panel B. The median pre-operative activity score was three (interquartile range (IQR) three to four) and this only improved slightly to four (IQR three to five) at three months post-operative, and then remained at this level for the duration of the 10-year follow-up.

Kneeling ability

The self-reported ability to kneel at each assessment time is presented in Table 3. The majority of patients were unable to kneel or had much difficulty kneeling, and this remained consistent over the duration of follow-up.

**Table 3:** Difficulty patients experienced when kneeling at each assessment time (%)

	Pre-op	3 months	1 year	2 year	3 year	5 year	7 year	10 years
Unable to kneel	52	43	39	44	45	46	46	58
With much difficulty	30	16	28	26	24	21	23	17
With a little difficulty	15	14	14	17	16	17	16	16
Can kneel easily	2	1	4	5	5	8	6	5
Not tried	1	26	15	9	10	8	9	4

American Knee Society Score

Median AKSS knee scores are presented in Figure 3, panel C. There was a large improvement in the median scores in the first three months, from 39 (IQR 29-49) pre-operatively to 77 (IQR 62-88) at three months post-operative. From three months to 10 years, there continued to be a small but gradual improvement to a median score of 93 (IQR 84-96) at 10 years post-operative.

Complications

Two early and three late deep infections occurred and were treated with revision surgery. Superficial wound infections were experienced by 13 patients during the early post-operative period, with nine patients requiring treatment with antibiotics. Three patients had aseptic loosening that required revision surgery. Three patients experienced periprosthetic fractures; these were treated with cannulated screws, supracondylar nailing, and open reduction and internal fixation. Eleven patients reported severe pain during the follow-up period. Seven patients reported swelling of the knee; investigation and treatment strategies included aspiration (three patients), physiotherapy (two patients) and no treatment (two patients). Six patients reported sensations of instability; three patients were revised for malalignment, one patient had physiotherapy, one patient had a brace and one patient required no treatment. Stiffness was reported by 18 patients. Of the 10 patients reporting an inadequate range of motion in the first three months post-operative, five patients had a manipulation under anaesthetic, one had a brace, one had physiotherapy and three patients had no treatment. Of the eight patients who reported stiffness beyond three months post-operative, three had a manipulation under anaesthetic, two had physiotherapy, one had removal of heterotrophic ossification and two had no treatment.

Survivorship

**Figure 4:**
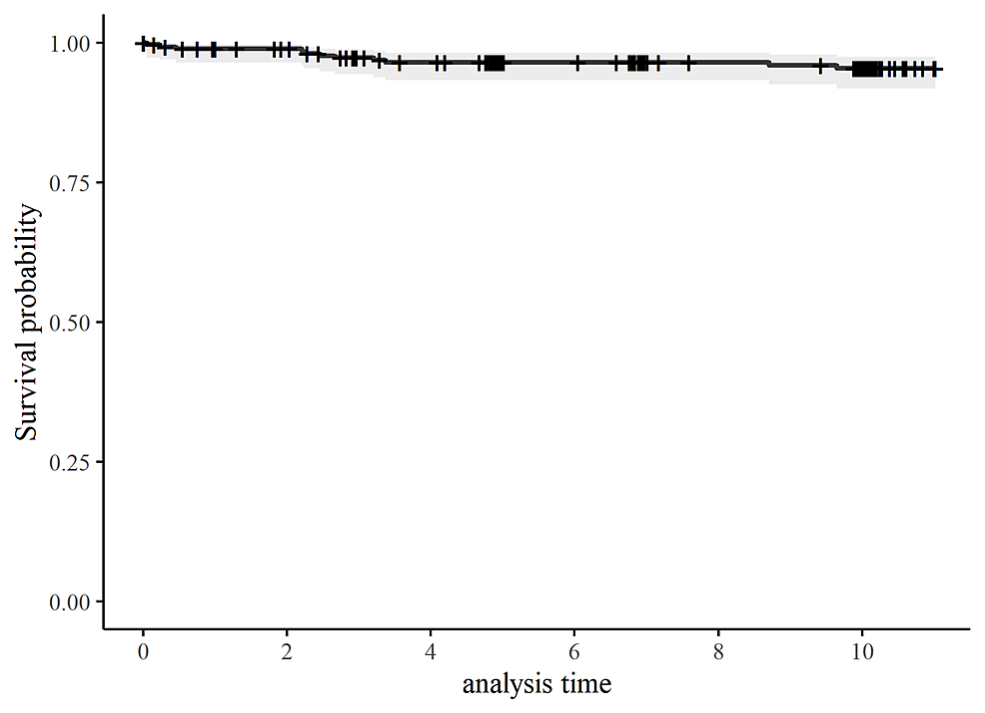
Survivorship of the Triathlon knee replacement

By 10 years post-operative, 11 (4.14%) patients had had their primary Triathlon TKR revised. Reasons for revision included infection (five patients), aseptic loosening (three patients) and malalignment (three patients). Survivorship with all-cause revision of the TKR as the endpoint was 95.4% (95% confidence interval 91.8-97.5) at 10 years post-operative (Figure 4).

## Discussion

Research has found that different prosthetic designs and brands can influence outcomes after TKR [24]. The National Joint Registry for England, Wales, Northern Ireland and the Isle of Man reported that 65 different brands of total condylar knee prostheses were used in primary TKR during 2018 [25]. Given this diversity, research is needed on the different brands of prostheses to monitor patient outcomes and generate evidence to aid decisions around the choice of implant. Findings from this cohort study confirmed our hypothesis that the Triathlon TKR results in good outcomes and survivorship up to 10 years post-operative.

Previous studies have evaluated the early and mid-term outcomes of the Triathlon TKR [12-15]. However, given that over 80% of TKRs can last up to 25 years [26], longer-term outcomes need to be evaluated. To the authors’ knowledge, only one previous cohort study has reported on patient-reported outcomes and survivorship up to 10 years after Triathlon TKR [16]. This study, based in Scotland, reported similar results to our study; however, we assessed a broader range of patient-reported outcomes, collected the AKSS and had more regularly post-operative assessments to gain more in-depth insight into long-term outcome trajectories. This previous study found that outcomes, assessed using the Oxford Knee Score, demonstrated the most improvement in the first six months, after which outcomes plateaued up to 10 years post-operative [16]. Our study further elaborates on this finding to provide evidence that the initial improvement in patient-reported pain, function, stiffness and knee-related quality of life mainly occurs in the first three months after surgery. While satisfaction ratings were high after TKR, little improvement was observed in activity levels and kneeling ability after surgery, which supports findings from previous studies [8,27] and highlights the need for research to evaluate interventions to improve these outcomes. The 10-year Kaplan-Meier survivorship with all-cause revision as the endpoint for the Triathlon TKR of 95.4% (95% confidence interval 91.8-97.5) is consistent with revision rates reported in the National Joint Registry (3.4%; 95% confidence interval 3.2-3.6) [1] and Australian Orthopaedic Association National Joint Replacement Registry (3.8%; 95% confidence interval 3.6-4.0) [28], and survivorship rates in the Finnish Arthroplasty Register (94%; 95% confidence interval 93-95) [29].

This study has strengths and weaknesses that should be considered when interpreting the results. Recruitment of patients was from one hospital, and therefore the findings may have limited external validity. The study recruitment rate was low (29%), likely due to the study design requiring participants to consent to multiple assessments over a 10-year follow-up period. However, the age and gender characteristics of participants were similar to the characteristics of patients recorded in the National Joint Registry [1], suggesting the sample is broadly representative of the wider population receiving TKR. We did not collect information on aspects of care that may have influenced the generalisability of the results, including surgical approach, anaesthetic management, and rehabilitation protocol; these were at the discretion of the operating surgeon. We did not collect radiographs as part of the study follow-up protocol, and therefore we were unable to conduct a radiographic evaluation of aseptic loosening. Follow-up rates were good, with 10-year questionnaires completed by 83% of patients who were still alive and had not had revision surgery. However, it is possible that the patients who did not complete the follow-up questionnaires had poorer outcomes than those who did complete the questionnaires. The strengths of the study design included a robust evaluation of outcomes using both patient-reported outcomes and a clinical assessment and a longitudinal study design with regular follow-up.

## Conclusions

Our results suggest that the Triathlon TKR results in excellent patient outcomes and survivorship up to 10 years post-operative and are comparable to other published research on the Triathlon knee prosthesis. Large improvements in pain, function, stiffness and quality of life occur in the first three months after TKR, followed by small improvement up to one year post-operative and then a plateauing of outcomes up to 10 years post-operative. There was little change in average activity levels and kneeling ability with TKR. These findings add to the limited evidence base on the long-term outcomes of the Triathlon TKR and can be used to inform decision-making by surgeons and patients around the choice of implant.
